# Rhythmic beta-frequency TMS over human right parietal cortex strengthens visual size illusions

**DOI:** 10.3758/s13423-025-02649-x

**Published:** 2025-02-06

**Authors:** Xue Han, Chao Wang, Lihong Chen

**Affiliations:** 1 Research Center of Brain and Cognitive Neuroscience, Liaoning Normal University, Dalian, 116029 China; 2 Key Laboratory of Brain and Cognitive Neuroscience, Dalian, 116029 Liaoning Province China

**Keywords:** Ebbinghaus illusion, Ponzo illusion, Superior parietal lobule, TMS, Beta oscillation

## Abstract

Rhythmic brain activity has been proposed to structure visual processing. Here we investigated the causal contributions of parietal beta oscillations to context-dependent visual size perception, which is indicated by the classic Ebbinghaus and Ponzo illusions. On each trial, rhythmic transcranial magnetic stimulation (TMS) was applied over the left or right superior parietal lobule in a train of five pulses at beta frequency (20 Hz). Immediately after the last pulse of the stimulation train, participants were presented with the illusory configuration, and performed a size-matching task. The results revealed that right parietal stimulation significantly increased the magnitudes of both size illusions relative to control vertex stimulation, whereas the illusion effects were unaffected with left parietal stimulation. Moreover, the stimulation effect was not observed with right parietal TMS at theta frequency (5 Hz). The findings clearly demonstrate the functional relevance of beta oscillations for the implementation of cognitive performance, supporting the causal contribution of parietal cortex to the processing of visual size illusions.

## Introduction

Human visual size perception is highly context dependent. For instance, an object appears larger when surrounded by several small items than by large ones (termed the Ebbinghaus illusion), or when placed at an apparently far location relative to a near location (termed the Ponzo illusion). Both size illusions are associated with the occipital cortex by showing that the illusion magnitudes are negatively correlated with the surface area of the primary visual cortex (V1) (Schwarzkopf et al., 2011; Schwarzkopf & Rees, 2013). Previous literature has suggested that the processing of the Ebbinghaus illusion takes place earlier in the visual pathway than that of the Ponzo illusion, as demonstrated by the fact that when the central target and its surrounds are simultaneously presented to different eyes, the Ebbinghaus illusion is significantly reduced, whereas the Ponzo illusion is unaffected (Song et al., 2011), and when the surrounds are rendered invisible, the Ebbinghaus illusion persists, whereas the Ponzo illusion disappears (Chen et al., 2018).

Converging evidence suggests that both size illusions rely on feedback projections from high- to low-level visual regions (Fang et al., 2008; Wu et al., 2023), and the parietal cortex is a candidate area for providing the feedback signals. The parietal and occipital cortex are naturally connected to allow for transmission of top-down attention signals (Silver & Kastner, 2009), and parietal transcranial magnetic stimulation (TMS) can increase neural activity in the early visual areas (Blankenburg et al., 2010; Koivisto et al., 2017; Ruff et al., 2008). Moreover, the parietal cortex is involved in object size perception (Harvey et al., 2015; Plewan et al., 2012) and visual size illusion (Song et al., 2017). Disruption of intrinsic parietal activity by repetitive TMS can temporally increase the Ebbinghaus illusion (Wu et al., 2023).

However, the potential mechanisms by which the parietal cortex affects the processing of visual size illusions are largely unexplored. Neural oscillations are proposed to be a fundamental mechanism supporting cognitive processes, with oscillations at different frequency bands playing distinct functional roles (Siegel et al., 2012). For instance, brain oscillations in the beta frequency range have been implicated in top-down processing and long-distance inter-area communication (Antzoulatos & Miller, 2016; Buschman & Miller, 2009; Marshall et al., 2015; Stanley et al., 2018; Stoll et al., 2016). Beta rhythmic TMS delivered over the right frontal eye field induces an increase of beta synchronization between this frontal area and ipsilateral parietal cortex and improves visual sensitivity of a low-contrast stimulus (Chanes et al., 2013; Quentin et al., 2015, 2016; Stengel et al., 2021; Vernet et al., 2019). When online rhythmic TMS at beta frequency is applied to the intraparietal sulcus, search accuracy is affected only during a conjunction search task that engages top-down attention but not during a feature search task which requires bottom-up attention (Riddle et al., 2019). Moreover, parietal beta oscillation has been found to be involved in perceptual grouping and perceptual discrimination (Battaglini et al., 2020; Romei et al., 2011; Ronconi et al., 2016; Zaretskaya & Bartels, 2015).

A short burst of rhythmic TMS not only locally entrains frequency-specific rhythm indicated by the pace of stimulation pulses at the target cortical area, but also synchronizes the stimulated target area with its interconnected region at the same frequency (Marshall et al., 2015). Therefore, we conjectured that when rhythmic beta stimulation was applied over the parietal cortex, it could increase beta oscillation, reflected in an increment of beta power (Battaglini et al., 2020) at the stimulated site, which might transmit to other connected areas, such as the occipital cortex, further shaping human perception. A functional magnetic resonance imaging (fMRI) study has found that the strength of the Ebbinghaus illusion is associated with an increase of feedback connection from the right parietal cortex to the extrastriate region (Chen et al., 2024). Thus, it is conceivable that rhythmic beta stimulation of the right parietal cortex would increase the size illusion effects relative to control vertex stimulation. To test whether the stimulation effect was frequency specific, we also applied rhythmic TMS at theta frequency over the right parietal region. Theta oscillations have been frequently observed at frontal and parietal regions and are involved in top-down processing (Karakaş, 2020; Rajan et al., 2019; Sauseng et al., 2008). Moreover, rhythmic TMS over the right parietal cortex at theta frequency can facilitate global visual processing (Romei et al., 2011). If the stimulation effect was beta-frequency specific, it would not be observed with theta TMS.

## Methods

### Participants

A total of 80 healthy right-handed participants (25 males; mean age 21.4 ± 2.3 years) took part in the study. Sixty-four took part in Experiment 1 (i.e., beta stimulation), 40 of them completed both size illusion tasks, another 12 performed only the Ebbinghaus illusion task, and another 12 conducted only the Ponzo illusion task. Sixteen participants took part in Experiment 2 (i.e., theta stimulation), and all of them performed both size illusion tasks. All participants only took part in one of the two experiments. The sample size was determined using G*Power software (Faul et al., 2007) to estimate the minimum sample size to reach 0.90 power for paired-sample t-test (two-tailed) with a mean effect size of 0.97 drawn from a previous similar study (Romei et al., 2011). All participants had normal or corrected-to-normal vision, with no metallic implants and no history of any neurological or psychiatric illness. They gave written informed consent prior to the study and were compensated for their time. None of the participants reported any adverse effects during the study. The protocol was approved by the institutional review board of Liaoning Normal University and adhered to guidelines of the Declaration of Helsinki.

### Stimuli and procedure

**Fig. 1:**
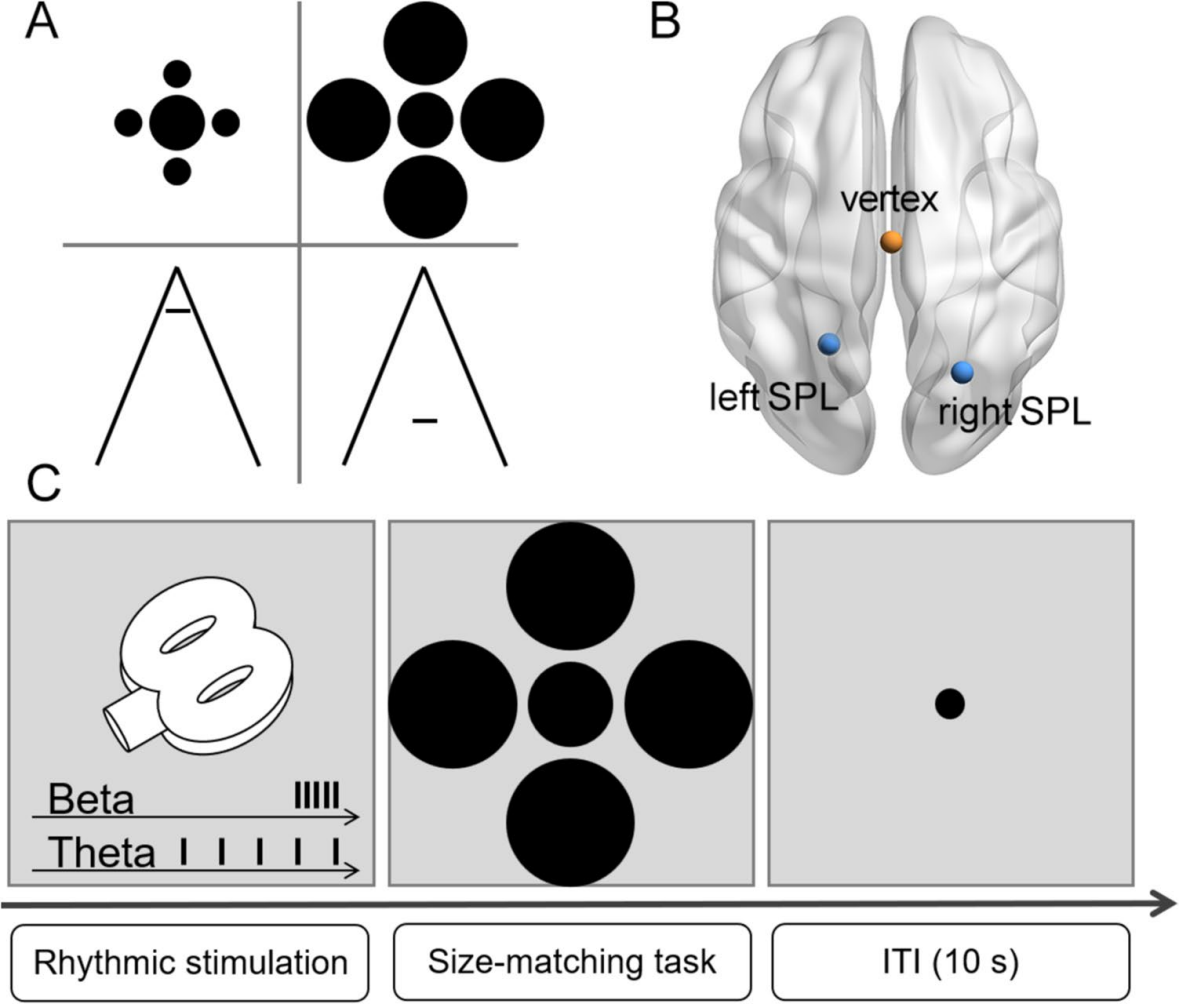
Experimental design. (**A**) Illustration of the Ebbinghaus and the Ponzo illusions. (**B**) Schematic presentation of stimulation sites. (**C**) Experimental procedure. On each trial, stimulation with a train of five pulses was applied to either the parietal or the vertex site. Immediately after the last pulse of the stimulation train, the illusory configuration was presented, and participants were required to perform the size-matching task. The inter-trial interval (ITI) was 10 s

Stimuli were displayed using MATLAB (The MathWorks, Natick, MA, USA) together with the Psychophysics Toolbox extensions (Brainard, 1997; Pelli, 1997). The Ebbinghaus configuration (Fig. 1A) comprised a central circle (diameter = 1.1°) surrounded by four large (diameter = 1.7°) or small (diameter = 0.6°) circles. The proximal distance between the central circle and the surrounding inducers was 0.14°. The initial size of a comparison circle was randomly selected from 0.8° to 1.4° in 0.057° steps on each trial. The Ponzo configuration consisted of a horizontal bar (1.1° × 0.2°) surrounded by a pair of converging lines (7.7° × 9.1°). The horizontal bar was presented either near or far from the vertex of the two converging lines (2.6° from screen center). The initial length of a comparison bar varied on each trial ranging from 0.8° to 1.4° in 0.057° steps. The central position of the illusory configurations was varied randomly within ± 0.3° of the screen center for each trial. All stimuli were black (RGB value: 0, 0, 0), and were presented on a gray background (RGB value: 128, 128, 128). Participants were positioned 57 cm from a computer screen (1,024 × 1,080 at 60 Hz) with their head position stabilized by a chin rest.

During the size illusion tasks, rhythmic TMS was applied over the left or right superior parietal lobule (SPL; Fig. 1B) in a train of five pulses at beta (20 Hz; Experiment 1) or theta (5 Hz; Experiment 2) frequency. Immediately after the last pulse of the stimulation train, an illusory configuration was presented at the screen center, and simultaneously a comparison stimulus was presented at the bottom (for the Ebbinghaus illusion task) or the right side of the screen (for the Ponzo illusion task; 8.6° from screen center; Fig. 1C). Participants were required to adjust the size of the comparison stimulus to match that of the target stimulus without time limit. The inter-trial interval was 10 s with a fixation point (0.28° × 0.28°) presented at the screen center. The vertex was selected as a control site to account for a non-specific TMS effect.

### Transcranial magnetic stimulation (TMS) protocol

A PowerMAG stimulator (Mag & More, Berlin, Germany) in combination with a figure 8-shaped coil (Double coil PMD70-pCool) was used to deliver stimulation. TMS Navigation (Visor 2, ANT-Neuro, Berlin, Germany) was employed to determine stimulation sites, to guide the placement and orientation of the coil and to allow online tracking for minimizing deviations from the optimal site of stimulation. Stimulation intensity was set at 90% of the individual resting motor threshold, which was defined as the minimal strength of stimulation capable of inducing a reliable twitch of the contralateral hand in at least five out of ten stimulations over the motor cortex (average threshold: 59.3% of maximum stimulator output). The stimulation frequency, intensity, and duration were well within safe limits.

Individual T1-weighted anatomical images (echo time = 2.9 ms, repetition time = 6.7 ms, field of view = 256 mm, matrix = 256 × 256, flip angle = 8°, spatial resolution = 1 × 1 × 1 mm^3^) were acquired using a 3.0 T scanner (MR-750, GE medical systems, Milwaukee, WI, USA). TMS was applied to either the left or right SPL (left MNI coordinates: x = −21, y = −52, z = 59; right MNI coordinates: x = 26, y = −62, z = 56), which have been found to be involved in visual size perception (Chen et al., 2022; Plewan et al., 2012). The vertex site (i.e., the highest point of the head, MNI coordinates: x = 1, y = −16, z = 76) was selected as a control condition (Soutschek et al., 2013). The coil was held tangentially to the skull and was oriented such that the coil center was overlaying the stimulation sites. To avoid any carry-over effects of TMS, the parietal stimulation and vertex stimulation were carried out on two separate days, with an interval of at least 1 week.

### Data analysis

The magnitude of the Ebbinghaus illusion was measured as the difference of the perceived size of the central target surrounded by small and large inducers relative to its physical size (%). The magnitude of the Ponzo illusion was calculated as the difference of the perceived length of the target bar presented at the upper and lower locations relative to its physical length (%). The illusion magnitude was entered into a repeated-measures ANOVA with stimulation location (SPL, vertex) as a within-subject factor and hemisphere (left or right) as a between-subject factor in Experiment 1. In Experiment 2, a paired-sample t-test (two-tailed) was performed. To draw definite conclusions about the viability of the null hypothesis in Experiment 2, we calculated the Bayes factor (BF) with Cauchy distribution (scale r = 1) to denote the likelihood of the null (H_0_) over the alternative (H_1_) hypothesis.

## Results

### Experiment 1: Beta TMS applied over left or right superior parietal lobule (SPL)

**Fig. 2:**
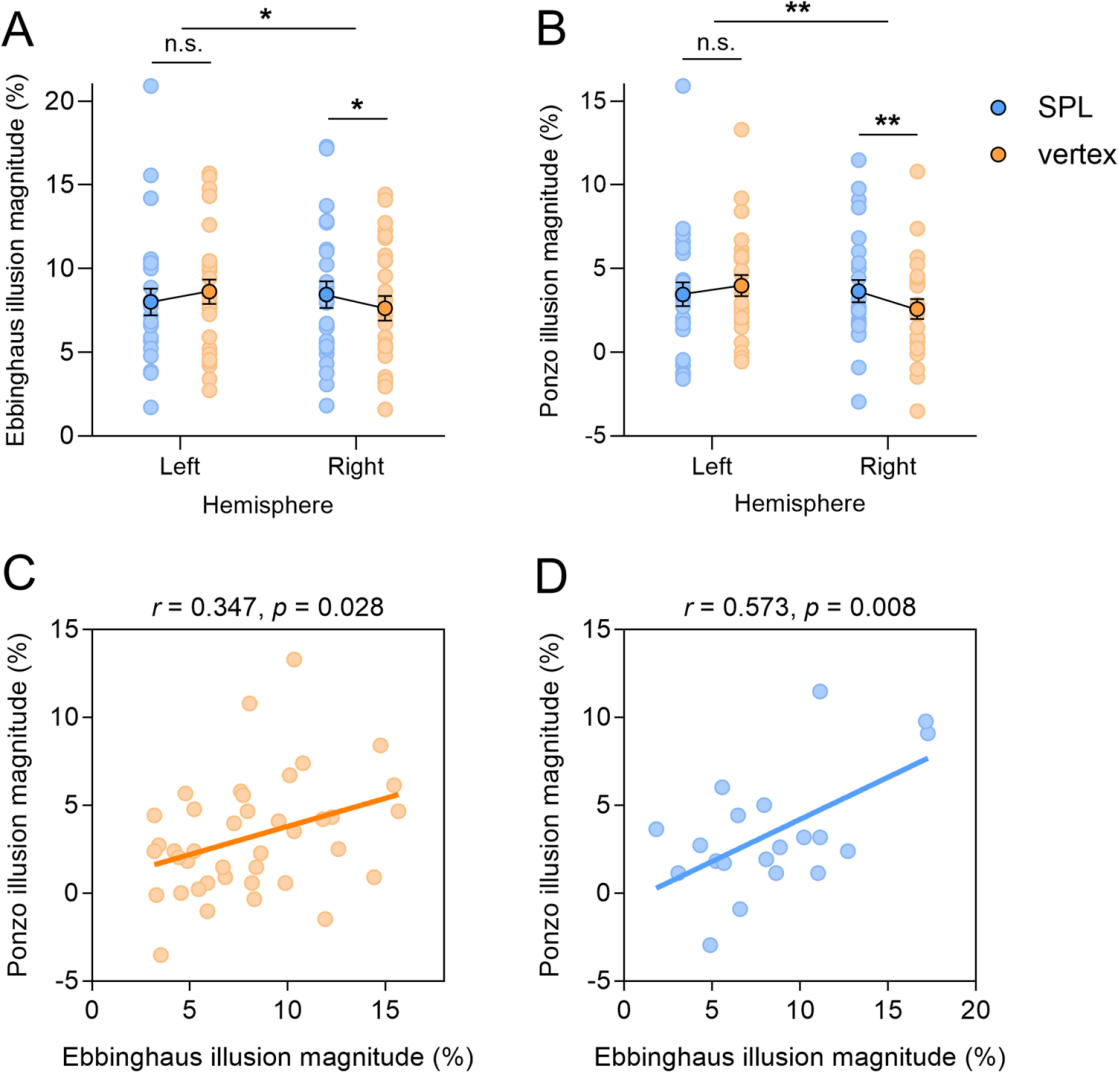
Results of Experiment 1. The magnitudes of (**A**) the Ebbinghaus and (**B**) the Ponzo illusions as a function of stimulation location and hemisphere. The correlation between the magnitudes of the Ebbinghaus and the Ponzo illusions for (**C**) the vertex stimulation and (**D**) the right superior parietal lobule (SPL) stimulation. Each dot represents data from one participant. Error bars represent one standard error of the mean. Asterisks indicate significance level: ∗ *p* < 0.05, ∗∗ *p* < 0.01

For the Ebbinghaus illusion task, the main effects of location (*F*(1,25) = 0.122, *p* = 0.729, η_p_^2^ = 0.002) and hemisphere (*F*(1,25) = 0.068, *p* = 0.795, η_p_^2^ = 0.001) failed to reach significance, but their interaction was significant (*F*(1,25) = 5.883, *p* = 0.019, η_p_^2^ = 0.105; Fig. 2A). Further analysis showed that, relative to control stimulation, right parietal stimulation significantly increased the illusion effect (*t*(25) = 2.160, *p* = 0.041, *d* = 0.424), whereas left parietal stimulation had a comparable effect on the illusion (*t*(25) = −1.355, *p* = 0.188, *d* = 0.266).

For the Ponzo illusion task, the main effects of location (*F*(1,25) = 0.916, *p* = 0.343, η_p_^2^ = 0.018) and hemisphere (*F*(1,25) = 0.490, *p* = 0.487, η_p_^2^ = 0.010) failed to reach significance, but their interaction was significant (*F*(1,25) = 7.893, *p* = 0.007, η_p_^2^ = 0.136; Fig. 2B). Further analysis revealed that, compared with control stimulation, right parietal stimulation significantly increased the illusion effect (*t*(25) = 3.129, *p* = 0.004, *d* = 0.614). However, this increment was not observed with left parietal stimulation (*t*(25) = −1.160, *p* = 0.257, *d* = 0.227).

The correlation between the magnitudes of the two size illusions was conducted for the 40 participants who performed both the Ebbinghaus and the Ponzo illusion tasks. All of them (*n* = 40) received vertex stimulation, with half (*n* = 20) receiving left SPL stimulation and the half (*n* = 20) right SPL stimulation. The results revealed a significant correlation during vertex stimulation (*r*(40) = 0.347, 95% confidence interval (CI) = [0.04, 0.59], *p* = 0.028; Fig. 2C). Notably, this positive correlation was strengthened with right SPL stimulation (*r*(20) = 0.573, 95% CI = [0.18, 0.81], *p* = 0.008; Fig. 2D] rather than with left SPL stimulation (*r*(20) = 0.316, 95% CI = [−0.15, 0.67], *p* = 0.175). It should be noted that the correlation coefficients for right SPL and vertex stimulations were comparable (*p* = 0.161, one-tailed).

### Experiment 2: Theta TMS applied over the right SPL

**Fig. 3:**
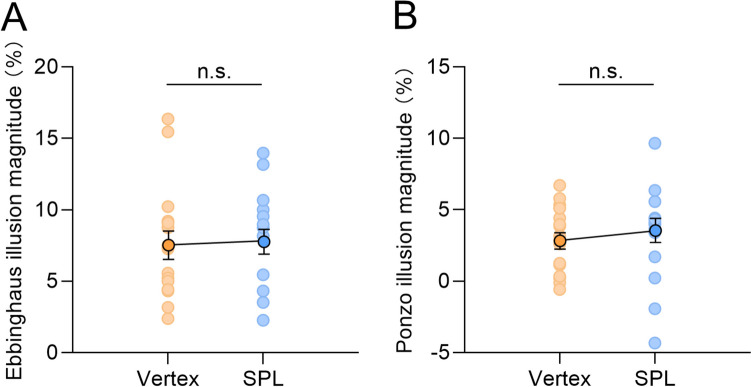
Results of Experiment 2. The magnitudes of (**A**) the Ebbinghaus and (**B**) the Ponzo illusions as a function of stimulation location. Each dot represents data from one participant. Error bars represent one standard error of the mean

Results of the paired-sample t-test showed that, compared with control vertex stimulation, right parietal stimulation at theta frequency had a comparable effect on both the Ebbinghaus (*t*(15) = 0.290, *p* = 0.776, *d* = 0.072, BF_01_ = 5.09; Fig. 3A) and the Ponzo (*t*(15) = 1.101, *p* = 0.288, *d* = 0.275, BF_01_ = 3.02; Fig. 3B) illusions.

## Discussion

By using a non-invasive brain stimulation technique, we showed that rhythmic parietal stimulation at beta frequency significantly increased the magnitudes of both the Ebbinghaus and the Ponzo illusions relative to control vertex stimulation. Notably, the stimulation effect was hemisphere asymmetric and frequency specific, i.e., it was observed when stimulation was applied to the right instead of the left parietal region, and at beta frequency rather than at theta frequency.

There is growing evidence that the parietal cortex is involved in conscious visual perception. For instance, the structure property of the parietal cortex, such as gray matter density, correlates with percept duration of bistable stimulus, and TMS over the parietal cortex can alter percept duration compared to control vertex stimulation (Kanai et al., 2010, 2011). Moreover, the neuromodulation effect on binocular rivalry is primarily observed with right rather than left parietal stimulation (Carmel et al., 2010; Zaretskaya et al., 2010). The amplitude of low-frequency fluctuation of the right SPL in a resting state predicts the magnitude of visual size illusions, and the forward connectivity strength from right V1 to right SPL negatively correlates with the strength of the Ebbinghaus illusion, and the self-connection strength in the right SPL negatively correlates with the strength of the Ponzo illusion (Wu et al., 2023). In line with and extending the above findings, the present study revealed that online rhythmic stimulation of right SPL at 20 Hz significantly increased the magnitude of visual size illusions. Notably, the stimulation-related changes were not observed with theta stimulation. Previous studies have found that when low-frequency (such as 1 Hz) TMS was applied over the right SPL, the neuromodulation effect is short-lived for the Ebbinghaus illusion (Wu et al., 2023), and is absent for the Müller-Lyer illusion (Mancini et al., 2011). The aforementioned findings suggest that the contribution of right SPL to visual size perception varies as a function of the frequency of neural oscillations.

Our study confirms a right lateralized involvement of the parietal cortex during conscious perception. Oscillating the activity of right rather than left SPL altered the perceived size of objects, establishing a causal role for this region in the control of context-dependent visual size perception. The dominance of the right hemisphere was further indicated by the fact that the correlation between the magnitudes of the two size illusions was strengthened with right instead of left parietal stimulation. The weakened correlation with left parietal stimulation could be due to interhemispheric competition (Hilgetag et al., 2003; Plow et al., 2014; Szczepanski & Kastner, 2013). It has been found that inhibitory stimulation of the left intraparietal sulcus by repetitive TMS decreases the activity of the stimulated site, whereas it increases the activity of its homologue region in the right hemisphere (Plow et al., 2014). We therefore speculated that left parietal stimulation might increase cortical excitability of the left parietal region, and simultaneously inhibit that of the right parietal region. Our findings could not provide direct evidence in favor of this hypothesis. Thus, the exact neural mechanisms need further exploration.

Given the interactive nature of brain areas, the effects of TMS are not limited to the targeted site but also spread to other short- and long-distance connected regions (Gallotto et al., 2022). Therefore, it is conceivable that parietal TMS not only affects the local function of parietal region, but also alters its functional interactions with remote but interconnected early visual cortex that is involved in visual size perception. The connectivity between the parietal and occipital cortex has been found to be involved in conscious visual perception. For instance, TMS applied to the intraparietal sulcus has been shown to modulate early visual cortical activity, and stronger effects were observed with right parietal TMS (Capotosto et al., 2012; Ruff et al., 2009). Activity in the extrastriate cortex consistently covaries with responses in the parietal cortex with a right hemispheric dominance during binocular rivalry (Lumer & Rees, 1999). The intrinsic connectivity from V1 to the right parietal cortex is associated with the strength of the Ebbinghaus illusion (Wu et al., 2023). Moreover, an object with a large perceived size increases the feedback connectivity from right parietal cortex to the extrastriate region relative to a physically identical object with a small perceived size (Chen et al., 2024). The present findings further suggest that the feedback signals from parietal to occipital cortex involved in visual size perception could transmit via inter-regional beta oscillations, which have been implicated in top-down modulation.

Beta neuronal oscillations have been suggested to be the natural rhythm of parietal cortex and are associated with top-down attention (Di Dona et al., 2024; Di Dona & Ronconi, 2023). In particular, beta-frequency TMS to intraparietal sulcus decreases search accuracy during the conjunction search task that engages top-down attention (Riddle et al., 2019). Increased beta activity in parietal regions is observed during interhemispheric binding of ambiguous visual motion (Costa et al., 2017). Short rhythmic stimulation can directly entrain a local oscillation at the targeted site (Thut et al., 2011). For instance, rhythmic TMS or transcranial alternating current stimulation (tACS) over the prefrontal cortex at beta frequency can preferably entrain endogenous beta oscillations at the stimulated site (Draganov et al., 2023; Hanslmayr et al., 2014). Therefore, in the current study, rhythmic TMS over the right parietal cortex at beta frequency might entrain parietal beta activity and further project modulatory signals to the occipital cortex to shape visual perception and performance.

In summary, our findings reveal increased context sensitivity (i.e., larger size illusion effect) with stimulation of right SPL at beta instead of theta frequency, elucidating the causal involvement of the right parietal cortex in context-dependent visual size perception, thus contributing to an emerging understanding of this region’s critical role in conscious awareness.

## Data Availability

Not applicable.
